# Intergenerational transmission of parental risky health behaviors in Chinese children: Are there socioeconomic status differences?

**DOI:** 10.3389/fmed.2022.842817

**Published:** 2023-01-09

**Authors:** Zexuan Yu, Wen Qin, Jiajia Li

**Affiliations:** ^1^Center for Health Management and Policy Research, School of Public Health, Cheeloo College of Medicine, Shandong University, Jinan, China; ^2^Department of Biostatistics, Brown University School of Public Health, Providence, RI, United States; ^3^Infirmary of Shandong University, Jinan, China; ^4^NHC Key Lab of Health Economics and Policy Research, Shandong University, Jinan, China

**Keywords:** intergenerational transmission, risky health behaviors, socioeconomic status, socioeconomic differences, CHNS

## Abstract

**Background:**

Risky health behaviors in childhood, including smoking, alcohol consumption, and having a poor diet, are the major sources of non-communicable diseases in adulthood. This study aimed to examine how parents affect children's risky health behaviors and whether intergenerational transmission differs based on socioeconomic status (SES).

**Methods:**

Data were extracted from the 1991–2015 China Health and Nutrition Survey (CHNS). Smoking (*n* = 5,946), alcohol consumption (*n* = 7,821), and sugar-sweetened beverages (SSBs) consumption (*n* = 3,537) were used as proxies for risky health behaviors in children. A binary choice model for panel data with a random-effect specification was employed to examine whether risky health behaviors can be transmitted from parents to their children. Subsequently, we conducted a seemingly unrelated estimation test (SUEST) to explore the differences in parental transmission between the different SES groups.

**Results:**

We found strong intergenerational persistence of smoking, alcohol drinking, and SSBs drinking behaviors, except for the mothers' smoking behavior. Mothers had a greater influence on children's alcohol drinking and SSBs drinking behaviors than fathers both in urban and rural areas and in different SES groups. The intergenerational transmission of SSBs drinking behavior exhibited a decreasing trend with increasing SES for both urban and rural families. In urban areas, mothers' alcohol drinking behavior has a decreasing trend with increasing education level, occupation, and income; however, in rural areas, the influence of mothers' alcohol drinking behavior occurred in the same direction with increasing education level and occupation type. In rural areas, the influence of fathers' drinking and smoking behaviors on children appears to mostly increase with increasing SES. Meanwhile, the influence of such behaviors among urban fathers would decrease with increasing SES.

**Conclusion:**

Parents' behaviors and SES can influence the initiation of risky health behaviors in their offspring. Thus, to promote healthy behaviors, policymakers can introduce health education programs for parents, particularly for those living in rural areas and with a low SES.

## 1. Introduction

Risky health behaviors, such as smoking, drinking alcohol, and having a poor diet, are major potential causes of death (1–6) and are often initiated in childhood and tend to persist into adulthood (5, 7–11). Thus, it is of great significance to prevent them earlier in life as the disease progresses (5, 12). However, the prevalence of smoking, alcohol drinking, and having unhealthy diets, such as sugar-sweetened beverages (SSBs), is substantial among children and adolescents. In 2018, ~43 million children aged 13–15 years used tobacco (13), and ~155 million adolescents were current drinkers globally (14). Investigations from the United States and China have found that over 60% of children and adolescents consume SSBs daily (15–18). Although the dangers of tobacco and alcohol are well-known (1, 10, 11, 13, 14), SSBs are dangerous because they often contain caffeine and sugar, which can be addictive. Caffeine addiction is a well-known problem (19, 20), but it has received more attention in recent years. Some animal-based studies have not only revealed similarities between added sugars and substance abuse in binge eating, craving, tolerance, and withdrawal (21) but also some have confirmed similar addictive characteristics in adolescents (22). In addition, some human neuroimaging studies have shown that high sugar intake activates neural circuits and reward systems like those of substance abuse (23, 24). Long-term sugar consumption can lead to obesity and diabetes, among other serious consequences (15–17).

Family is a key environment that influences children's behavior. As children's first teachers and socializing agents, parents' negative health behaviors can act as a bad model for their children (25–28). In addition, parental socioeconomic status (SES) variables, including educational attainment, income, and occupational status, together with parenting styles, constitute the home environment in which children's behaviors are embedded (25, 29–39).

There have already been many studies on the relationship between SES and children's risky health behaviors; however, whether SES differences affect the intergenerational transmission of unhealthy behaviors has not been sufficiently clarified. Previous studies have focused on parents' own behavior and the relationship between SES and parenting styles, which provide a reference for our own research proposition (26, 27, 29–31, 40–44). According to Cockerham's Health Lifestyle Theory (45), high-SES parents not only avoid the transmission of their own negative health behaviors but also their gentle parenting style helps to develop self-control in their children (45). In contrast, low-SES families tend to adopt strict, punitive, and authoritarian parenting styles, leading to children's poor self-control and making them more likely to emulate their parents' risky health behavior (28–31, 33, 34, 39, 46–52). In other words, the intergenerational transmission of risky health behaviors is likely to be in reverse to the SES gradient. However, other research studies showed that with increasing SES, the intergenerational transmission of risky parental health behaviors becomes increasingly apparent (35, 37, 53–55). Yu and Abler (54) proposed that higher education in rural China is often associated with more social activities, and with cigarettes and wine being more accessible. Wu (56) confirmed Yu's conclusion regarding the association between education and alcohol drinking. Furthermore, a Belgian study found that higher-educated mothers tended to have higher workloads and thus spent less time with their children, making them more vulnerable to risky health behaviors (53). Therefore, the contribution of this study is not only to measure the intergenerational transmission effect of these risky health behaviors with panel data including nine waves spanning 15 years but also to further measure whether there are SES differences in the intergenerational transmission effect based on Cockerham's health lifestyle theory with seemingly unrelated estimation test (SUEST). Urban and rural differences are also considered.

In the hope of adding up-to-date evidence to previous cross-sectional studies and using the longitudinal database from the China Health and Nutrition Survey (CHNS), this study aimed to examine how parents affect children's risky health behaviors. China, a developing country with rapid economic growth, is quite different from developed countries that have been studied earlier in this field (28, 31, 33, 39). Thus, the characteristics of this research proposal may be different, and the research conclusions can provide a reference for other developing countries. As a developing country, and unlike developed countries like the United States, China's laws do not make it clear that children's smoking and drinking behaviors are illegal. Without such legal restrictions, children's behaviors depend more on family constraints, and hence identifying the effects of intergenerational transmission of these risky health behaviors is more critical (25, 57–61). Moreover, considering that adults often have difficulty making behavioral changes to addictive behaviors, it may be more effective to prevent and reduce risky health behaviors in children from the perspective of reducing intergenerational transmission. Therefore, we further explored whether intergenerational transmission differed by parental SES.

## 2. Methods

### 2.1. Data

The primary database used in this study was the CHNS. The CHNS is an ongoing nationwide cohort project in China with 10 available waves from 1989 to 2015. These areas are representative and diverse in terms of a wide range of socioeconomic factors (including income, education, and employment) and other related demographic, health, and nutritional factors. As only individuals aged between 20 and 45 years were surveyed in 1989, we excluded the baseline data, and only used data from 1991 to 2015 in the analysis, singling out smoking, alcohol drinking, and SSB drinking as proxies for risky health behaviors in children aged <18 years. We excluded samples with outliers and missing data, leaving 5,946 observations in the smoking group, 7,821 observations in the alcohol drinking group, and 3,537 observations in the SSBs drinking group. After matching the parent's ID with the child's ID, we obtained 24,573 observations in total: 4,609 observations in 1991, 4,140 in 1993, 3,577 in 1997, 3,312 in 2000, 1,972 in 2004, 1,583 in 2006, 1,424 in 2009, 1,782 in 2011, and 2,174 in 2015. Due to the existence of missing values in the dependent and control variables, we had to drop records containing missing values. Finally, we obtained 5,946 observations in the sample of children's smoking behavior, 7,821 observations in the sample of children's alcohol drinking behavior, and 3,537 observations in the sample of children's SSBs drinking behavior.

### 2.2. Measures

The main dependent variables in this study were children's smoking, alcohol drinking, and SSB drinking behaviors. Smoking was assessed using the question, “Have you ever smoked?,” and was coded as 1 if the respondent answered “Yes.” Alcohol consumption was assessed based on the question, “Did you drink beer or any other alcoholic beverage?” and was coded as 1 if the respondent answered “Yes.” SSBs consumption was assessed based on the question, “Did you drink soft drinks or sugared fruit?” and coded as 1 if the respondent answered “Yes.” All risky health behaviors were answered by the respondents. We then linked answers from the parent questionnaires to those of their children.

The independent variables were the risky parental health behaviors of these children, which were also assessed based on the three questions above. To analyze how risky health behaviors are transmitted from parents to their children, we also included parental SES variables, including educational attainment (completed years of formal education in regular schools), household per capita income (RMB in 2015), and career type (manual labor/non-manual labor). Demographic variables, such as age (years) and sex (male/female), were also included. In addition, we controlled the area (categorized as Western: Guangxi, Guizhou, and Chongqing; Northeastern: Liaoning and Heilongjiang; Central: Henan, Hubei, and Hunan; Eastern: Jiangsu, Shandong, Beijing, and Shanghai) and wave (1991, 1993, 1997, 2000, 2004, 2006, 2009, 2011, and 2015) effects. The variable of education was transformed into a dichotomous variable, with >6 years of education being classified as a high level of education, and ≤ 6 years of education being classified as a low level of education.

### 2.3. Statistical analysis

Data analyses were conducted using STATA/SE 14.0. Descriptive statistics for both parental and children's risky health behaviors, including smoking, alcohol use, and drinking SSBs, were reported as proportions, with corresponding chi-square tests to examine whether these behaviors were statistically significant for transmission from parents to their children. Parental SES and demographic variables were also estimated as proportions for categorical variables and means for continuous variables; chi-square tests for dichotomous variables and *t*-tests for continuous variables were conducted, and *p*-values were reported.

To investigate whether parental risky health behaviors could be transmitted and how these behaviors were transmitted, we adopted a binary choice model for panel data with random-effect specification after conducting the Hausman test (*p*_*smoking*_= 1.000, *p*_*alcohol*_= 0.9043, *p*_*SSBs*_= 0.7745). Odds ratios (ORs) with their *p*-values are reported. The model is specified as follows:


(1) $$\begin{array}{l} ln\frac{P_{i,t}}{1-P_{i,t}}=\beta_{0}+\beta_{1}FatherBehavior_{i,t}+\beta_{2}MotherBehavior_{i,t}\\ \;+\sum_{j=1}^{4}\beta_{3j}FatherSES_{ij,t}+\sum_{j=1}^{4}\beta_{4j}MotherSES_{ij,t}\\ \;+\beta_{5}Area_{i,t}+\beta_{6}Wave_{i,t}+\beta_{7}Gender_{i,t}+\beta_{8}Age_{i,t}\\ \;+u_{i,t} \end{array}$$

where *P*_*i,t*_ represents the probability of children's smoking, alcohol drinking, and SSBs drinking behaviors; *FatherBehavior*_*i,t*_/*MotherBehavior*_*i*_ indicates whether the *child*_*i*_'s father/mother had this kind of risky health behavior, including smoking, alcohol drinking, and SSBs drinking; *FatherSES*_*ij,t*_/*MotherSES*_*ij,t*_ represents the *child*_*i*_'s father/mother's SES; *Wave*_*i,t*_ indicates the time dummies to explore the dynamic evolution from 1993 to 2015; *Area*_*i,t*_ indicates the region dummies to explore the region's effects on children's risky health behaviors; *Gender*_*i,t*_ and *Age*_*i,t*_ represent the *child*_*i*_'s gender and age individually. *u*_*i,t*_ represent the individual effects on the child. We used the model above to analyze the total sample, the urban sample, and the rural sample.

To understand the influence of different SES variables on the intergenerational transmission of risky health behaviors, we grouped urban and rural parents according to their education level, income, and occupation type, used model (1) for regression in different subgroups, and drew a bar chart with confidence intervals. Regarding parental education level, we divided parents into low-level ( ≤ 6 years) and high-level (>6 years) education subgroups. Regarding income, those with an income equal to or lower than the average were included in the low-income subgroup, whereas those with an income higher than the average were included in the high-income subgroup. Finally, we divided occupations into manual labor and non-manual labor subgroups and then conducted a subgroup analysis. To test the differences in the coefficients *FatherBehavior*_*i,t*_ and *MotherBehavior*_*i*_ among different subgroups, a SUEST was used.

## 3. Results

### 3.1. Descriptive analysis

The variables used in this study are displayed in Table 1 and include the entire sample, as well as the risky health behavior and non-risky health behavior samples.

**Table 1:** Descriptive statistics.

**Variables**	**Smoking**				**Alcohol drinking**				**SSBs drinking**			

	**Total**	**No**	**Yes**	*p* ^a^	**Total**	**No**	**Yes**	*p* ^a^	**Total**	**No**	**Yes**	*p* ^a^
	**(*****n*** = **5,946)**	**(*****n*** = **5,686)**	**(*****n*** = **260)**		**(*****n*** = **7,821)**	**(*****n*** = **7,318)**	**(*****n*** = **503)**		**(*****n*** = **3,537)**	**(*****n*** = **631)**	**(*****n*** = **2,906)**	
**Father's**				0·000				0·000				0·000
**behavior**,												
***n*** **(%)**												
No	1,749 (29·41)	1,712 (30·11)	37 (14·23)		2,311 (29·55)	2,212 (30·23)	99 (19·68)		2,418 (68·36)	571 (90·49)	1,847 (63·56)	
Yes	4,197 (70·59)	3,974 (69·89)	223 (85·77)		5,510 (70·45)	5,106 (69·77)	404 (80·32)		1,119 (31·64)	60 (9·51)	1,059 (36·44)	
**Mother's**				0·089				0·000				0·000
**behavior**,												
***n*** **(%)**												
No	5,792 (97·41)	5,543 (97·49)	249 (95·77)		6,852 (87·61)	6,490 (88·69)	362 (71·97)		1,984 (56·09)	538 (85·26)	1,446 (49·76)	
Yes	154 (2·59)	143 (2·51)	11 (4·23)		969 (12·39)	828 (11·31)	141 (28·03)		1,553 (43·91)	93 (14·74)	1,460 (50·24)	
**Father's**				0·000				0 063				0 004
**education**												
Less or equal to 6 years	2,334 (0.39)	2,203 (0.39)	131 (0.50)		3,007 (0.38)	2,794 (0.38)	213 (0.42)		743 (0.21)	159 (0.25)	584 (0.20)	
More than 6 years	3,612 (0.61)	3,483 (0.61)	129 (0.50)		4,814 (0.62)	4,524 (0.62)	290 (0.58)		2,794 (0.79)	472 (0.75)	2,322 (0.80)	
**Mother's**				0·000				0.058				0·000
**education**												
	3,393 (0.57)	3,205 (0.56)	188 (0.72)		4,394 (0.56)	4,091 (0.56)	303 (0.60)		1,161 (0.33)	267 (0.42)	894 (0.31)	
	2,553 (0.43)	2,481 (0.44)	72 (0.28)		3,427 (0.44)	3,227 (0.44)	200 (0.40)		2,376 (0.67)	364 (0.58)	2,012 (0.69)	
Income (Inflated to 2015, LN), mean (SD)	8·26 (1·17)	8·27 (1·17)	8·26 (1·21)	0·0185	8·08 (1·11)	8·06 (1·11)	8·31 (1·03)	0·0000	8·73 (1·29)	8·32 (1·36)	8·82 (1·25)	0.0000
**Father's**				0·666				0·788				0·000
**job**, ***n*** **(%)**												
Manual labor	3,507 (58·98)	3,357 (59·04)	150 (57·69)		4,678 (59·81)	4,380 (59·85)	298 (59·24)		1,936 (54·74)	434 (68·78)	1,502 (51·69)	
Non-manual labor	2,439 (41·02)	2,329 (40·96)	110 (42·31)		3,143 (40·19)	2,938 (40·15)	205 (40·76)		1,601 (45·26)	197 (31·22)	1,404 (48·31)	
**Mother's**				0·516				0·453				0·000
**job**, ***n*** **(%)**												
Manual labor	3,775 (63·49)	3,605 (63·40)	170 (65·38)		5,050 (64·57)	4,733 (64·68)	317 (63·02)		2,027 (57·31)	442 (70·05)	1,585 (54·54)	
Non-manual labor	2,171 (36·51)	2,081 (36·60)	90 (34·62)		2,771 (35·43)	2,585 (35·32)	186 (36·98)		1,510 (42·69)	189 (29·95)	1,321 (45·46)	
Age, mean (SD)	14·98 (1·96)	14·89 (1·94)	16·93 (1·31)	0·0000	12·71 (4·50)	12·50 (4·53)	15·72 (2·61)	0·0000	11·87 (3·58)	12·19 (3·76)	11·80 (3·54)	0·0133
**Gender**,				0·000				0·000				0·099
***n*** **(%)**												
Male	3,115 (52·39)	2,862 (50·33)	253 (97·31)		4,120 (52·68)	3,723 (50·87)	397 (78·93)		1,949 (55·10)	329 (52·14)	16,20 (55·75)	
Female	2,831 (47·61)	2,824 (49·67)	7 (2·69)		3,701 (47·32)	3,595 (49·13)	106 (21·07)		1,588 (44·90)	302 (47·86)	1,286 (44·25)	
**Area**,				0·000				0·000				0·001
***n*** **(%)**												
Western (Ref·)	1,921 (32·31)	1,845 (32·45)	76 (29·23)		2,704 (34·57)	2,523 (34·48)	181 (35·98)		956 (27·03)	197 (31·22)	759 (26·12)	
Northeastern	868 (14·60)	831 (14·61)	37 (14·23)		1,014 (12·97)	964 (13·17)	50 (9·94)		709 (20·05)	143 (22·66)	566 (19·48)	
Central	1,829 (30·76)	1,695 (29·81)	134 (51·54)		2,368 (30·28)	2,177 (29·75)	191 (37·97)		1,100 (31·10)	185 (29·32)	915 (31·49)	
Eastern	1,328 (22·33)	1,315 (23·13)	13 (5·00)		1,735 (22·18)	1,654 (22·60)	81 (16·10)		772 (21·83)	106 (16·80)	666 (22·92)	
**Wave**,				0·025				0·000				0·000
***n*** **(%)**												
1991 (Ref·)	1,177 (19·79)	1,104 (19·42)	73 (28·08)		3,009 (38·47)	2,862 (39·11)	147 (29·22)					
1993	1,015 (17·07)	973 (17·11)	42 (16·15)		1,243 (15·89)	1,163 (15·89)	80 (15·90)					
1997	1,008 (16·95)	973 (17·11)	35 (13·46)		982 (12·56)	915 (12·50)	67 (13·32)					
2000	576 (9·69)	545 (9·58)	31 (11·92)		563 (7·20)	517 (7·06)	46 (9·15)					
2004	669 (11·25)	647 (11·38)	22 (8·46)		669 (8·55)	629 (8·60)	40 (7·95)		1,112 (31·44)	286 (45·32)	826 (28·42)	
2006	441 (7·42)	421 (7·40)	20 (7·69)		439 (5·61)	402 (5·49)	37 (7·36)		845 (23·89)	200 (31·70)	645 (22·20)	
2009	358 (6·02)	343 (6·03)	15 (5·77)		358 (4·58)	322 (4·40)	36 (7·16)		706 (19·96)	80 (12·68)	626 (21·54)	
2011	424 (7·13)	410 (7·21)	14 (5·38)		426 (5·45)	386 (5·27)	40 (7·95)		874 (24·71)	65 (10·30)	809 (27·84)	
2015	278 (4·68)	270 (4·75)	8 (3·08)		132 (1·69)	122 (1·67)	10 (1·99)					

N.B.

a χ^2^ tests for dichotomous variables and *t*-tests for continuous variables.

The prevalence of smoking, alcohol consumption, and SSBs consumption in children was 4.37, 6.43, and 82.16%, respectively. Boys had significantly higher proportions of these three risky health behaviors than girls. Children who smoked and drank alcohol were significantly older than those who did not smoke and drink alcohol, while those drinking SSBs were significantly younger than those who did not drink SSBs.

Both fathers and mothers of smoking children had higher smoking rates, while the difference in mothers' smoking rates between smoking and non-smoking children was not significant. Both fathers and mothers of children who drank alcohol had a significantly higher rate of alcohol consumption than those who did not drink alcohol. Similarly, among children who drank SSBs, both fathers and mothers had a significantly higher ratio of drinking SSBs than the fathers and mothers of children who did not drink SSBs.

### 3.2. Logistic regression results

The results of the logistic regression for intergenerational transmission of risky parental health behaviors in Chinese children are presented in Table 2. In the total sample, after controlling for confounding variables, children who had a smoking father were ~240.9% more likely to smoke (*p* < 0.01) than children who had a non-smoking father. While the intergenerational transmission of fathers' smoking behavior was observed among rural children, the intergenerational transmission effect was even more pronounced among urban children; smoking fathers increased the probability of children smoking by 2,506% (*p* = 0.034). Considering the unreliability of the small sample size on mothers' smoking, maternal smoking transmission is not reported here.

**Table 2:** Results of random-effect logistic regression.

**Variables**	**Smoking**			**Alcohol Drinking**			**SSBs Drinking**		

	**All OR(SD)**	**Urban OR (SD)**	**Rural OR (SD)**	**All OR (SD)**	**Urban OR (SD)**	**Rural OR(SD)**	**All OR (SD)**	**Urban OR (SD)**	**Rural OR (SD)**
Father's behavior: Yes	3·409^***^	26·06^**^	2·835^***^	1·715^***^	2·194^***^	1·526^**^	2·573^***^	2·748^***^	2·508^***^
	(0·882)	(40·05)	(0·737)	(0·234)	(0·526)	(0·257)	(0·421)	(0·876)	(0·492)
Mother's behavior: Yes	1·784	76·22^**^	1·142	3·574^***^	3·147^***^	3·395^***^	3·592^***^	3·394^***^	3·917^***^
	(0·894)	(164·1)	(0·633)	(0·530)	(0·649)	(0·705)	(0·504)	(0·952)	(0·656)
Father's education	0·961	1·229	0·954	1·007	1·026	0·999	1·005	0·912^*^	1·030
	(0·0314)	(0·251)	(0·0317)	(0·0197)	(0·0338)	(0·0257)	(0·0201)	(0·0451)	(0·0244)
Mother's education	0·938^**^	0·706^*^	0·985	0·992	0·959	1·001	1·037^**^	1·105^**^	1·016
	(0·0295)	(0·135)	(0·0328)	(0·0180)	(0·0304)	(0·0249)	(0·0182)	(0·0495)	(0·0212)
Income	0·962	2·281	0·919	1·160^**^	1·235	1·071	1·165^***^	1·126	1·162^***^
	(0·0846)	(1·278)	(0·0801)	(0·0768)	(0·158)	(0·0823)	(0·0468)	(0·0973)	(0·0552)
Father's job: Non-manual labor	1·329	9·559	1·399	0·913	0·998	1·130	1·384^**^	0·811	1·556^**^
	(0·338)	(13·53)	(0·374)	(0·136)	(0·226)	(0·234)	(0·207)	(0·251)	(0·294)
Mother's job: Non-manual labor	0·788	5·384	0·454^**^	0·976	0·897	0·547^**^	1·244	1·210	1·008
	(0·210)	(8·854)	(0·162)	(0·149)	(0·226)	(0·140)	(0·189)	(0·415)	(0·204)
Age	2·531^***^	10·13^***^	2·299^***^	1·429^***^	1·454^***^	1·408^***^	1·001	1·077^**^	0·975
	(0·233)	(4·846)	(0·209)	(0·0376)	(0·0608)	(0·0480)	(0·0144)	(0·0354)	(0·0160)
Gender: Male	84·16^***^	135,565^***^	53·80^***^	4·533^***^	3·982^***^	5·002^***^	1·251^**^	1·193	1·268^**^
	(42·32)	(405,173)	(27·75)	(0·635)	(0·817)	(0·944)	(0·129)	(0·280)	(0·148)
Area	Yes	Yes	Yes	Yes	Yes	Yes	Yes	Yes	Yes
Wave	Yes	Yes	Yes	Yes	Yes	Yes	Yes	Yes	Yes
Constant	1·93e-10^***^	0^***^	3·50e-09^***^	2·52e-05^***^	1·97e-05^***^	4·69e-05^***^	0·287^***^	0·579	0·343^**^
	(4·05e-10)	(0)	(6·96e-09)	(1·85e-05)	(2·81e-05)	(4·37e-05)	(0·115)	(0·643)	(0·167)
Observations	5,946	1,682	4,264	7,821	2,124	5,697	3,537	1,063	2,474

*** *p* < 0.01,

** *p* < 0.05,

* *p* < 0.1.

Similarly, to smoking fathers, fathers who drank alcohol increased the possibility of alcohol drinking in their children by 71.5% (*p* < 0.01) in the total sample, 119.4% (*p* = 0.01) in the urban sample, and 52.6% (*p* = 0.012) in the rural sample. Mother's alcohol consumption increased the possibility of alcohol consumption by 257.4% (*p* < 0.01) in the total sample, 214.7% (*p* < 0.01) in the urban sample, and 239.5% (*p* < 0.01) in the rural sample.

Likewise, in the total sample, children whose fathers drank SSBs were ~161.2% more likely to drink SSBs (*p* < 0.01) than children whose fathers did not drink SSBs. Indeed, SSBs drinking could increase the likelihood of children consuming SSBs by 259.2% (*p* < 0.01). A similar intergenerational transmission of this behavior was observed in both urban and rural children. Gender plays an important role in the intergenerational transmission of risky health behaviors. Boys were ~8,316, 353.3, and 25.1% more likely to smoke, drink alcohol, and drink SSBs, respectively, than girls. Furthermore, age also plays an important role in the intergenerational transmission of smoking and alcohol consumption. Similar effects were observed in both urban and rural children.

Various parental SES variables were also shown to significantly affect children's behavior. A higher parental per capita income was shown to make both urban and rural children more likely to drink alcohol and SSBs. However, the effects of educational attainment and occupational status were not consistent or even opposite between urban and rural areas and between parents.

### 3.3. Subgroup analysis

**Figure 1:**
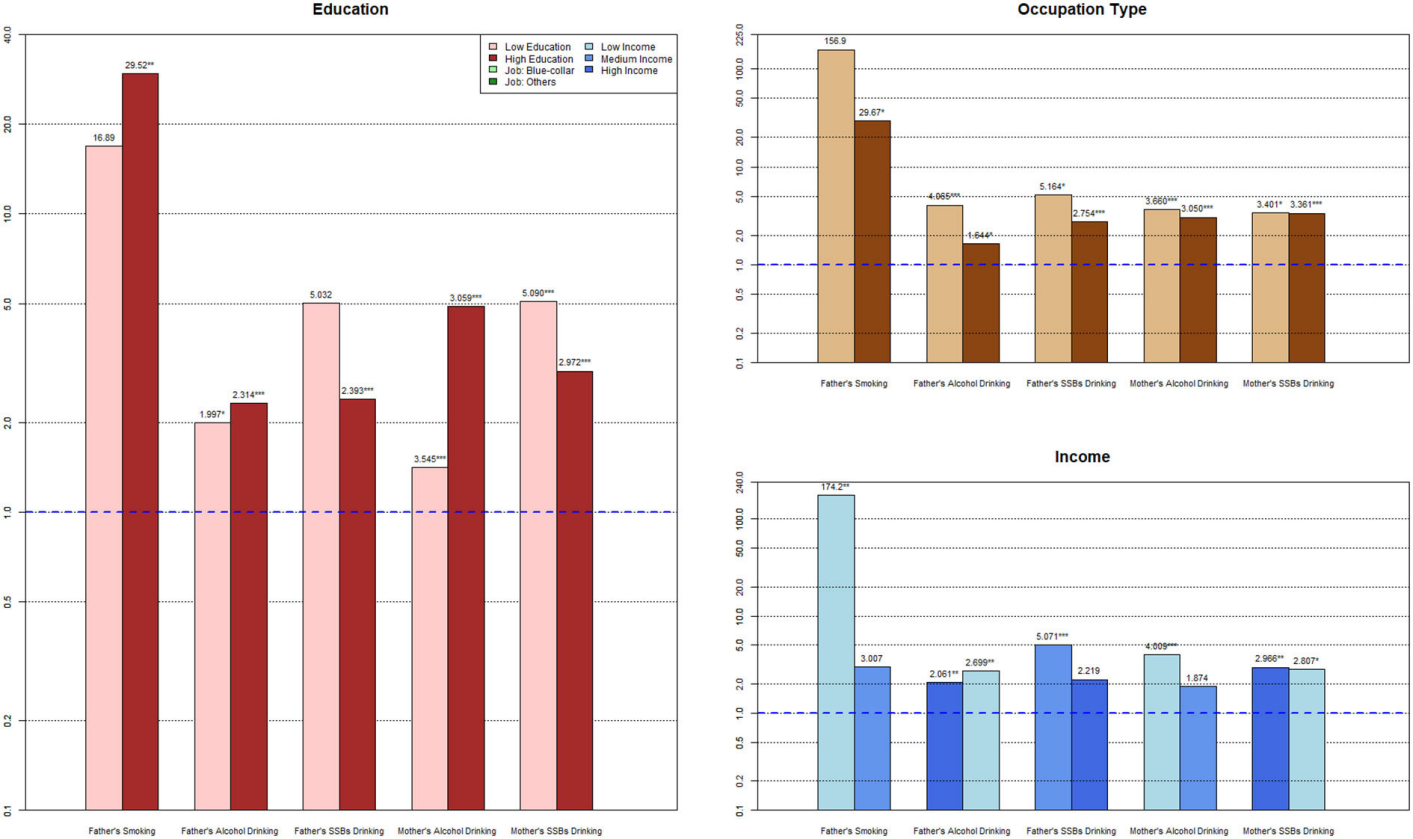
Subgroup analysis for urban families.

**Figure 2:**
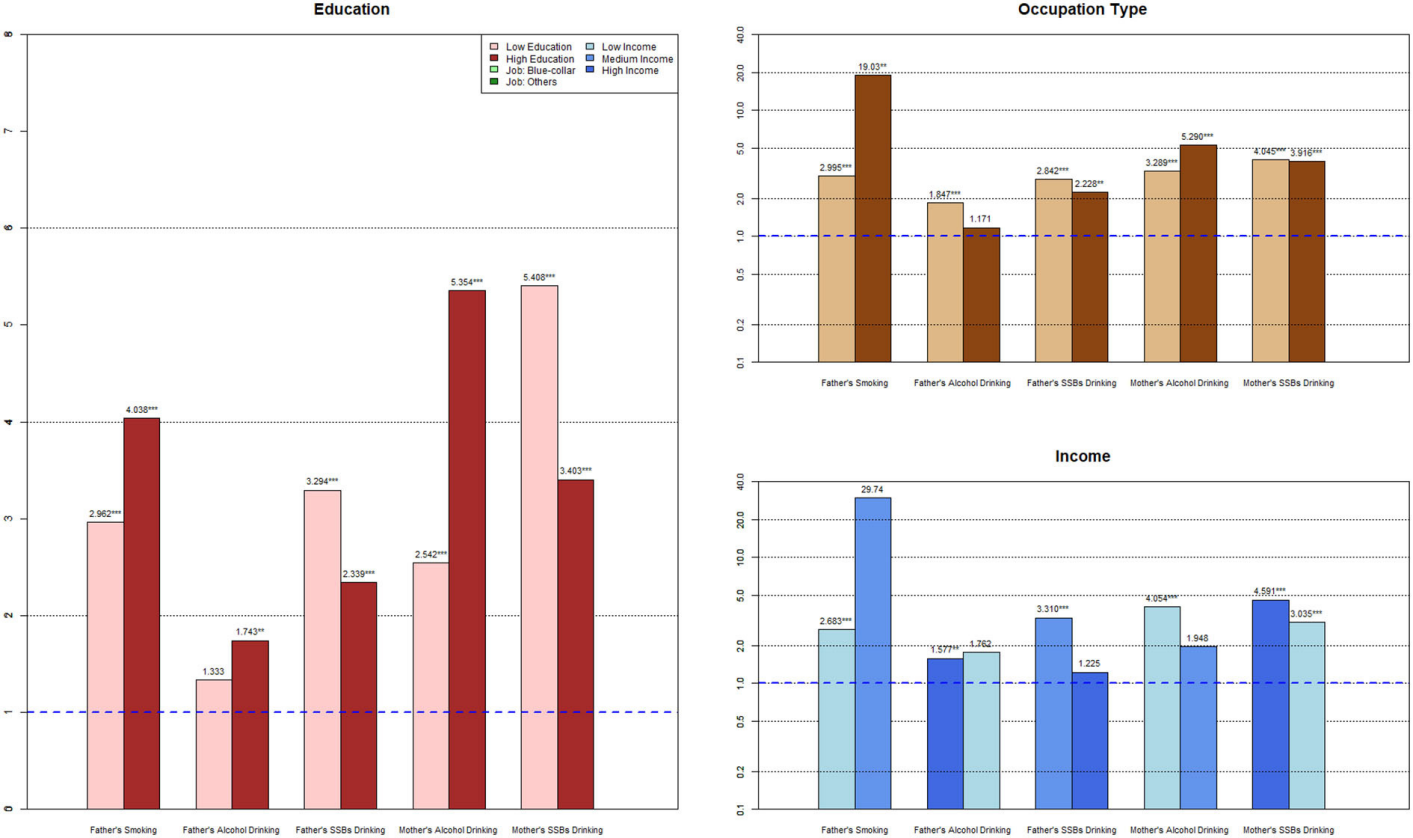
Subgroup analysis for rural families.

To further clarify the intergenerational transmission of urban and rural parental risky health behaviors between SES groups, the results of the subgroup analysis and SUEST are shown in Table 3, Figures 1, 2. Considering the unreliability of the subgroup analysis due to the small sample size of mothers' smoking, maternal smoking transmission between different SES groups is not reported here.

**Table 3:** Subgroup analysis of intergenerational transmission with different SES.

**Variables**	**Urban**			**Rural**		

	**OR (SD)**	**Suest test**	**Observations**	**OR (SD)**	**Suest test**	**Observations**
**Smoking**						
Low father education	16·89 (48·27)	χ^2^ = 0·04, *p =* 0·8468	187	2·962^***^ (1·065)	χ^2^ = 0·76, *p =* 0·3818	1,858
High father education	29·52^**^ (48·64)		1,206	4·038^***^ (2·033)		2,406
Low mother education	0·232 (0·795)		336	1·202 (0·641)		2,724
High mother education	9,533^**^ (37,501)		912	–		825
Father's job: Manual labor	156·9 (512·1)	χ^2^ = 1·42, *p =* 0·2340	631	2·995^***^ (0·874)	χ^2^ = 1·95, *p =* 0·1625	2,782
Father's job: Non-manual labor	29·67^*^ (58·76)		957	19·03^**^ (25·52)		1,482
Mother's job: Manual labor	9,089 (57,695)		418	0·961 (0·532)		3,229
Mother's job: Non-manual labor	7·763 (15·59)		585	–		1,023
Low income: Father's effect	174·2^**^ (376·2)	χ^2^ = 0·27, *p =* 0·6013	478	2·683^***^ (0·738)	χ^2^ = 0·01, *p =* 0·9279	3,271
High income: Father's effect	3·007 (2·479)		411	29·74 (64·33)		744
Low income: Mother's effect	0·00132 (0·00538)		478	1·501 (0·807)		3,271
High income: Mother's effect	8·549^**^ (8·145)		411	–		744
**Alcohol drinking**						
Low father education	1·997^*^ (0·836)	χ^2^ = 0·00, *p =* 0·9499	587	1·333 (0·346)	χ^2^ = 0·84, *p =* 0·3584	2,420
High father education	2·314^***^ (0·699)		1,537	1·743^**^ (0·389)		3,277
Low mother education	3·545^***^ (0·966)	χ^2^ = 0·99, *p =* 0·3189	829	2·542^***^ (0·658)	χ^2^ = 2·90, *p =* 0·0888	3,565
High mother education	3·059^***^ (0·902)		1,295	5·354^***^ (1·939)		2,132
Father's job: Manual labor	4·065^***^ (1·896)	χ^2^ = 4·81, *p =* 0·0283	849	1·847^***^ (0·410)	χ^2^ = 1·54, *p =* 0·2142	3,792
Father's job: Non-manual labor	1·644^*^ (0·456)		1,238	1·171 (0·328)		1,905
Mother's job: Manual labor	3·660^***^ (1·201)	χ^2^ = 0·45, *p =* 0·5016	651	3·289^***^ (0·820)	χ^2^ = 0·69, *p =* 0·4048	4,399
Mother's job: Non-manual labor	3·050^***^ (0·780)		1,473	5·290^***^ (1·947)		1,298
Low income: Father's effect	2·061^**^ (0·648)	χ^2^ = 1·25, *p =* 0·2639	1,348	1·577^**^ (0·306)	χ^2^ = 1·00, *p =* 0·3710	4,707
High income: Father's effect	2·699^**^ (1·314)		523	1·762 (0·650)		792
Low income: Mother's effect	4·009^***^ (1·135)	χ^2^ = 0·51, *p =* 0·4742	1,348	4·054^***^ (0·967)	χ^2^ = 0·41, *p =* 0·5235	4,707
High income: Mother's effect	1·874 (0·875)		523	1·948 (0·956)		792
**SSBs drinking**						
Low father education	5·032 (8·027)	χ^2^ = 0·35, *p =* 0·5524	125	3·294^***^ (1·397)	χ^2^ = 1·91, *p =* 0·1669	604
High father education	2·393^***^ (0·789)		924	2·339^***^ (0·536)		1,870
Low mother education	5·090^***^ (3·170)	χ^2^ = 1·45, *p =* 0·2290	205	5·408^***^ (1·650)	χ^2^ = 5·39, *p =* 0·0203	933
High mother education	2·972^***^ (0·943)		835	3·403^***^ (0·706)		1,541
Father's job: Manual labor	5·164^*^ (4·799)	χ^2^ = 0·46, *p =* 0·4966	429	2·842^***^ (0·701)	χ^2^ = 3·93, *p =* 0·0473	1,507
Father's job: Non-manual labor	2·754^***^ (1·067)		634	2·228^**^ (0·738)		967
Mother's job: Manual labor	3·401^*^ (2·372)	χ^2^ = 0·35, *p =* 0·5533	350	4·045^***^ (0·798)	χ^2^ = 3·19, *p =* 0·0739	1,677
Mother's job: Non-manual labor	3·361^***^ (1·050)		713	3·916^***^ (1·236)		797
Low income: Father's effect	5·071^***^ (2·621)	χ^2^ = 4·43, *p =* 0·0354	341	3·310^***^ (0·868)	χ^2^ = 13·33, *p =* 0·0003	1,312
High income: Father's effect	2·219 (1·286)		410	1·225 (0·407)		885
Low income: Mother's effect	2·966^**^ (1·253)	χ^2^ = 0·54, *p =* 0·4610	341	4·591^***^ (1·043)	χ^2^ = 11·51, *p =* 0·0007	1,312
High income: Mother's effect	2·807^*^ (1·498)		410	3·035^***^ (0·875)		885

*** *p* < 0.01,

** *p* < 0.05,

* *p* < 0.1.

#### 3.3.1. Subgroup analysis for urban families

The higher the father's education level, the more significant the intergenerational transmission of smoking (OR_LowEdu_ = 16.89, OR_HighEdu_ = 29.52) and alcohol drinking (OR_LowEdu_ = 1.997, OR_HighEdu_ = 2.314); however, the OR of SSBs drinking dropped from 5.302 to 2.393, in the low education vs. the high education group, respectively. From low education level to high education level, the OR of the mother's alcohol drinking behavior decreased from 3.545 to 3.509, and the OR of maternal SSBs drinking decreased from 5.090 to 2.972, with insignificant differences in coefficients between the maternal low education and high education groups.

Fathers in the manual labor group had a more significant intergenerational transmission of smoking (OR_Manuallabor_ = 156.9, OR_Non − manuallabor_ = 29.67), alcohol drinking (OR_Manuallabor_ = 4.065, OR_Non − manuallabor_ = 1.644), and SSBs drinking (OR_Manuallabor_ = 5.164, OR_Non − manuallabor_ = 2.754) than fathers in the non-manual labor group. Non-manual labor mothers showed significantly fewer intergenerational transmission effects of alcohol drinking (OR_Manuallabor_ = 3.660, OR_Non − manuallabor_ = 3.050) and SSBs drinking (OR_Manuallabor_= 3.401, OR_Non − manuallabor_ = 3.361).

With an increase in income, fathers' intergenerational transmission effect of smoking and SSBs consumption became weaker and insignificant (OR_LowInc_ = 174.2, OR_HighInc_ = 3.007; OR_LowInc_ = 5.071, OR_HighInc_ = 2.219). In contrast, the transmission effect of fathers' wine drinking behavior became stronger (OR_LowInc_ = 2.061, OR_HighInc_ = 2.699) as income increased. Both mother's alcohol drinking behavior (OR_LowInc_ = 4.009, OR_HighInc_ = 1.874) and SSBs drinking behavior (OR_LowInc_ = 2.966, OR_HighInc_ = 2.807) decreased from the low-income to the high-income group.

#### 3.3.2. Subgroup analysis for rural families

With the rise in paternal education level, intergenerational transmission of fathers' smoking (OR_LowEdu_ = 2.962, OR_HighEdu_ = 4.038) and alcohol drinking (OR_LowEdu_ = 1.333, OR_HighEdu_ = 1.743) increased, but the OR of fathers' SSBs drinking behavior dropped from 3.294 to 2.339. The OR of highly educated mothers' alcohol drinking behavior increased from 2.542 to 5.354, and that of maternal SSBs drinking reduced from 5.408 to 3.403, with significant coefficient differences (*p*_alcoholofSUEST_ = 0.0888; *p*_ssbsofSUEST_ = 0.0203) than mothers in the low-education group.

Smoking in fathers in non-manual labor jobs had a stronger effect on children's formation of this behavior (OR_Manuallabor_ = 2.995, OR_Non − manuallabor_ = 19.03) than in fathers in manual labor jobs. Meanwhile, –labored fathers were less connected with children's alcohol drinking (OR_Manuallabor_ = 1.847, OR_Non − manuallabor_ = 1.171) and SSBs drinking (OR_Manuallabor_ = 2.842, OR_Non − manuallabor_ = 2.228). Furthermore, mothers in non-manual labor jobs showed significantly lower effects on children's alcohol drinking (OR_Manuallabor_ = 3.289, OR_Non − manuallabor_ = 5.290) and SSBs drinking (OR_Manuallabor_ = 4.045, OR_Non − manuallabor_= 3.916) than mothers in manual labor jobs.

With an increase in income, the father's intergenerational transmission effect of smoking became stronger (OR_LowInc_ = 2.683, OR_HighInc_ = 29.74), with a similar effect being observed for fathers' wine drinking behavior (OR_LowInc_ = 1.577, OR_HighInc_ = 1.762). On the contrary, the influence of fathers' SSBs drinking behavior on their children's behavior went through a process of weakening as income increased (OR_LowInc_ = 3.310, OR_HighInc_ = 1.225), and the coefficient difference was significant (*p-values*_ofSUEST_ = 0.0003). Both mother's alcohol drinking behavior (OR_LowInc_ = 4.054, OR_HighInc_ =1.948) and SSBs drinking behavior (OR_LowInc_ = 4.591, OR_HighInc_ = 3.035) decreased from the low-income to the high-income group (*p*_ssbsofSUEST_ = 0.0007), whereas other subgroups did not.

## 4. Discussion

This study has three central findings: (1) risky health behaviors had significant intergenerational transmission effects; (2) the intergenerational transmission of mothers' alcohol consumption and SSBs drinking behavior was greater than that of fathers; and (3) the influence of SES on intergenerational transmission in urban areas was different from that of rural children. For addictive behaviors, such as alcohol drinking and smoking, intergenerational transmission mostly exhibited a decreasing trend with increasing SES in urban families but increased with increasing SES for rural families. However, the influence of parental SSBs drinking behaviors showed a consistent decreasing trend with increasing SES for both urban and rural families.

This study showed that, consistent with many previous findings (25, 29–39, 46, 48–50, 62), risky parental health behaviors have a significant risk of being transmitted to their children. It is usually easy for children to imitate their parents' behaviors even if they are negative or unhealthy (25–28, 63, 64), and, therefore, parents' words and deeds are very important in preventing risky health behaviors in children.

Importantly, differences between paternal and maternal influences were also observed. Contrary to many previous studies (25, 29, 32, 33, 65, 66), we found that fathers' smoking behavior had a more significant effect on leading children's smoking behavior than mothers. This may be because the sample size of maternal smokers in this study was too small to make good statistical inferences than previous studies. However, when it came to alcohol drinking and SSBs drinking, the impact of both these behaviors on children was significantly higher from mothers than from fathers, which was consistent with some previous research (29, 49, 61). This may be because mothers generally spend more time with their children than fathers. Therefore, children could be more affected by their mother's behavior than their father's (29, 49, 61).

The role of parental SES in the intergenerational transmission of risky health behaviors in urban and rural children was not exactly the same. In general, there was a significant decrease in the intergenerational transmission of SSBs drinking behavior with increasing SES: the risky health behavior transmission effect of high-SES parents was weaker than that of low-SES parents, and SUEST showed that only the education level of fathers in rural areas had no significant differences between groups, while only fathers' income level had significant differences between groups in urban areas. We also noted that the intergenerational transmission effect of urban mothers' drinking behavior tended to decrease when all three SES variables increased, but rural mothers' alcohol drinking behavior had a similar changing trend with the rise in education level and occupational class. However, SUEST showed that only the education levels of mothers in rural areas differed significantly between the subgroups. A similar situation occurred among fathers: rural fathers' risky health behaviors mainly appeared to show a similar changing trend with SES, while urban fathers' smoking had a reverse changing trend with the rise in occupational type and income. However, SUEST showed that there were significant inter-subgroup differences in the occupation types of fathers in urban areas, but there were no significant differences between the other subgroups.

This reflected that SES had a dual influence on the intergenerational transmission of risky parental health behavior. On the one hand, higher SES means better family capital and better parenting style, which will prevent the formation of children's unhealthy behaviors and reduce the transmission of these behaviors from their parents (26, 27, 30, 41, 42, 49, 67–71). High-SES parents were usually well aware of the dangers of risky health behavior and were therefore inclined to discourage their children from these behaviors, while low-SES parents often did not care whether their children engaged in these behaviors or even engaged in these behaviors in front of their children, setting a bad example for their children and leading them to engage in these behaviors (27, 41, 42, 67, 70–72). Some facts support the standpoint that higher SES can promote people's health and healthy behaviors. In the health model presented by Grossman, more affluent families tend to spend more money on healthcare, such as better-quality medical care and healthy food (40, 68, 71). Well-educated parents are more inclined to adopt healthy behaviors, both for themselves and their children, so the incidence of risky health behaviors among children is lower (68, 69, 71). In addition to the fact that education can lead to a better knowledge of the importance of promoting healthy behaviors, there were also potential indirect effects, such as smoother ways to get a job, better affordability of health-improving goods, less stress, and better work environments due to high-SES parents also being exposed to healthier colleagues (35, 43, 71). However, lower-SES people may care less about their health and that of their family members, be less responsive to health promotion, receive less information about how to get healthy, and have limited access to health promotion services (34, 67, 70). Similar effects of low-SES have been observed in risky health behaviors, such as SSBs consumption, which mainly exists in children and adolescents.

On the contrary, however, higher SES can mean that parents will devote more time to their own careers to cope with higher workloads (53), more often ignoring messages they received, hiring nannies or, in Chinese traditional culture, asking for support from their own retired parents, who are often less educated, leading to the absence of family education. Children with high-SES parents tend to have more disposable pocket money, which makes it easier for them to access substances that pose health risks, such as SSBs and wine (73). All of these factors may increase the risk of unhealthy behaviors in children. If the degree of intergenerational transmission is more severe than that of rural and low SES parents, the effect of parents' actions is greater than the effect of their words, which may promote intergenerational transmission of risky health behaviors in children. This may be because high-SES parents tend to have a higher status in their children's minds, and children are more likely to imitate risky parental health behaviors (25, 74). In our sample, for traditional rational addictive behaviors such as smoking and alcohol drinking, higher parental occupation and education level could enhance intergenerational transmission, which is an example of the effect of their actions being greater than that of their words. This shows the necessity and importance of behavioral changes starting with the parents.

## 5. Conclusion

We observed that parents played an important role in the development of risky health behaviors in children. Risky parental health behaviors set a bad example for their children and tempt children to imitate their parents' behaviors. It is worth noting that urban areas, especially urban mothers, mostly reflected the positive effects of SES, whereas fathers, especially those in rural areas, reflected adverse effects. This suggests that we should pay more attention to fathers' behaviors and awareness of health education in rural areas and invest in the rearing of their children. In addition to persuading children to drop these behaviors, more attention should be paid to reducing intergenerational transmission.

## Data availability statement

The original contributions presented in the study are included in the article/Supplementary material, further inquiries can be directed to the corresponding authors.

## Ethics statement

Data for this study was from the China Health and Nutrition Survey (CHNS). CHNS was approved by the Institutional Review Committees of the University of North Carolina at Chapel Hill and the National Institute of Nutrition and Food Safety, Chinese Center for Disease Control and Prevention.

## Author contributions

ZY and JL conceptualized and designed the study, participated in explaining the data, and writing and revising the manuscript. ZY analyzed the data. WQ helped to revise the manuscript. All authors approved the final version of the paper and have directly accessed and verified the underlying data reported in the manuscript.
